# Evaluation of crystalline amino acids as potent stimulatory chemoattractants for the slipper lobster *Thenus orientalis*

**DOI:** 10.7717/peerj.15607

**Published:** 2023-10-20

**Authors:** Chui-Fen Teoh, Audrey Daning Tuzan, Annita Seok-Kian Yong, Kit-Shing Liew, Leong-Seng Lim, Hon-Jung Liew

**Affiliations:** 1 Borneo Marine Research Institute, Universiti Malaysia Sabah, Kota Kinabalu, Sabah, Malaysia; 2 Higher Institution Centre of Excellence (HICOE), Institute of Tropical Aquaculture and Fisheries, Universiti Malaysia Terengganu, Kuala Nerus, Terengganu, Malaysia; 3 Heilongjiang River Fisheries Research Institute, Chinese Academy of Fishery Sciences, Harbin, China

**Keywords:** Chemoattractant, Chemoreceptive behavior, Behavioral responses, Amino acids, Thenus orientalis

## Abstract

Intensive research on the effectiveness of chemoattractants has been widely explored to improve the feed qualities in expanding crustacean farming. Taste preferences in slipper lobster remained unknown despite their significant contribution to the lobster fisheries. Chemoattractants allow better performance in aquaculture species by increasing food attractiveness and palatability. Amino acids (AA) have been leading in previous research on crustacean feeding behavior. Given that slipper lobster possesses chemoreceptors to detect and orient towards food, this study investigated an approach to identify the AA with the most potent chemoattractant in eliciting a response from slipper lobster. Behavioral assays were performed to evaluate the responses of slipper lobster *Thenus orientalis* (carapace length, 52.34 ± 1.52 mm) on 15 crystalline AA and three derivatives of AA (DAA) at three concentrations between 10^−1^ and 10^−3^ M as test substances (TS). *Meretrix* sp. extract was used as a positive control and clean filtered seawater as a negative control. The behavioral responses of 14 *T. orientalis* were evaluated based on their antennular flicking rate, third maxillipeds activity, and substrate probing by the pereiopods. *T. orientalis* responded to the solutions of single AA down to a concentration of 10^−3^ M, excluding histidine and serine. The behavioral activity displayed by *T. orientalis* increased with the TS concentrations. L-glutamic acid monosodium salt monohydrate, betaine, and glycine solutions elicited the most behavioral responses, whereas histidine exhibited the lowest behavioral responses. Conclusively, L-glutamic acid monosodium salt monohydrate, betaine, and glycine can be potential chemoattractants for *T. orientalis*.

## Introduction

*Thenus orientalis* is commonly known as bugs, shovelnose, and sand lobsters and represents the most demanded species among Scyllaridae species (Latrouite, Booth & Jamieson, 2009). Scyllarid lobsters are among the major commercial interests as valuable seafood in Asia, Europe, and America (Rogers, Barnard & Johnston, 2010), and account for 8% of global lobster production (Radhakrishnan et al., 2019). Currently, *Thenus* spp. has been successfully bred and reared in a laboratory-scale settlement but no large-scale commercial aquaculture of scyllarid lobster species has been performed (Gopalakrishnan, Radhakrishnan & Phillips, 2019). Slipper lobster has not been extensively investigated following their relative insignificance in commercial fisheries operations until recently (Lavalli, Spanier & Grasso, 2007). Slipper lobster usually contributes to the by-catch of spiny lobster (Spanier & Lavalli, 2007), Australian prawn, scallop trawl (Johnston & Alexander, 1999), and other fisheries. Many fishermen have developed an interest in the farming of slipper lobsters given the high market value and decreased abundance of spiny lobsters (Spanier & Lavalli, 2007). Nonetheless, this shift has further endangered slipper lobsters to overexploitation due to insufficient knowledge of this species (Alborés, García-Soler & Fernández, 2019).

Aquaculture production of slipper lobster is an alternative approach to reducing pressure on overexploited wild stock. In Malaysia, live *Thenus* sp. (USD30/kg, live) has a lower market value compared to live *Panulirus* sp. (USD89/kg, live). Nevertheless, scyllarid lobster has a faster growth rate and can reach maturity within 400 days after hatching (Hart, 2009; Mikami, 2007; Vijayakumaran & Radhakrishnan, 2011). In contrast, a minimum of 365 days was required to raise pueruli *P. ornatus*, the planktonic stage of metamorphoses from the last phyllosoma instar (Guerao, Díaz & Abelló, 2006), to an acceptable legal size (300 g) (Tamm, 1980). Despite similar egg size (0.6–0.8 mm), the most valuable spiny lobster *P. ornatus* (120–150 days) with the shortest larval cycle among palinurid and homarid species (Barnard et al., 2011; Smith et al., 2009; Takei, 1977), has a more prolonged larval duration than slipper lobster (28 days) (Latrouite, Booth & Jamieson, 2009; Mikami & Greenwood, 1997; Smith et al., 2009). Furthermore, the egg incubation of each female *T. orientalis* may occur in more than one brood per year (Latrouite, Booth & Jamieson, 2009), which renders this species a fast-emerging candidate for aquaculture (Radhakrishnan et al., 2019).

The production of slipper lobster in captivity is challenging due to the lack of formulated feeds, and slipper lobsters were only fed on fresh mussels and fish (Alborés, García-Soler & Fernández, 2019). As traditional feeding with natural foods is not cost-effective, continuing this practice further exacerbates the degradation of the environment due to its low conversion efficiency (Radhakrishnan et al., 2019). The development of a practical diet is the critical and only solution for sustainable aquaculture. A previous preliminary experiment revealed that wild slipper lobster *T. orientalis* rejected formulated diet and relied on fresh *Meretrix* sp. Given that not all aquatic animals are ready to accept formulated diets, several studies on effective chemoattractants and feeding stimulants have been reported. A feeding stimulant triggers an animal to continue feeding once it starts to feed, while the chemoattractant triggers an animal to search and move toward the chemical source (Lavalli, Spanier & Grasso, 2007; Lim et al., 2021; Smith et al., 2005). Most studies on chemoattraction in crustaceans have demonstrated low molecular weight compounds, including amino acids, amines, nucleotides, and organic acids (Lee & Meyers, 1996). Amino acids are highly prevalent among these chemoattractants and act as effective attractants that are species-specific in lobsters (Carter & Steele, 1982; Johnson & Ache, 1978; Lim et al., 2021; Zimmer-Faust, 1987; Zimmer-Faust et al., 1984).

The detection of AA is critical in crustacean feeding behavior (Voigt & Atema, 1992). The binding sites of the chemoreceptors are designed specifically for specific molecules (Carr, Ache & Gleeson, 1987). *Homarus americanus* possessed chemoreceptor specificity to different AA in the antennular sensation (Shepheard, 1974). Voigt & Atema (1992) reported diverse chemoreceptor cell types in all the chemoreceptor organs in *H. americanus*, including antennae, antennules, walking legs, and maxillipeds. Chemoreceptive behaviors played a significant role in the feeding responses of crustaceans toward chemical stimuli (Lee & Meyers, 1996; Zimmer-Faust, 1987; Zimmer-Faust et al., 1984). Slipper lobster possesses chemoreceptors to detect and orient towards food using antennules (Lavalli, Spanier & Grasso, 2007). Nevertheless, there is no available information on the chemoreceptor responses of *T. orientalis* for a particular AA. Hence, initial chemosensory studies that tested individual chemicals were vital in identifying the chemicals that are most likely to stimulate physical attraction and feeding. These events provide the rationale to determine the potent stimulatory chemoattractant for *T. orientalis* by assessing the effectiveness of AA. This study also investigated the behavioral responses of *T. orientalis* exposed to three concentrations (10^−3^ M, 10^−2^ M, and 10^−1^ M) of amino acids.

## Materials & Methods

### Experimental animals

Wild-caught *T. orientalis* purchased from local fishermen at Sabah Fish Marketing, Kota Kinabalu, were acclimatized for a week in a blue plastic tank (190 cm L × 103 cm W × 100 cm H, surface area: 6.84 m^2^, 50 cm seawater depth, 1-ton water volume) with well-aerated water. Recirculating aquaculture system (RAS) was equipped with a sump tank, protein skimmer, and ultraviolet filters in the Crustacean Hatchery of Borneo Marine Research Institute. Direct sunlight into the acclimatized tank was reduced by covering the tank with a black sunshade garden mesh net (90%). During the acclimatizing period, the fresh squid *Loligo* sp. was hand-fed to each *T. orientalis* at 30% of the total biomass daily. After an hour, uneaten food and waste particles were removed daily through syphonation.

All procedures were approved by the Universiti Malaysia Sabah Animal Ethics Committee (AEC008/2022) for handling experimental animals and the Sabah Biodiversity Council approved research permits (JKM/MBS.1000-2/2 JLD.13 (96)) for conducting research in Sabah.

### Preparation of chemical stimuli

The behavioral assay consisted of three types of solutions: (a) the *Meretrix* sp. extract as the positive control, (b) the test substances (TS), comprising 15 AA and three derivatives of AA at three different concentrations (10^−1^ M, 10 ^−2^ M, and 10^−3^ M), and (c) the clean filtered seawater (SW) as the negative control. The *Meretrix* sp. was prepared weekly for the lobster conditioning and behavioral assay. This conditioning process was critical for acclimatizing the lobster to the feeding regimes with the presence of the *Meretrix* sp. extract (Liew et al., 2022). In preparing the *Meretrix* sp. extract, the flesh of the live *Meretrix* sp. was minced with a chopper and filtered through a one mm pore size sieve net to collect the homogenate. The 10% w/v aqueous extract of the *Meretrix* sp. was prepared by mixing 100 g of *Meretrix* sp. homogenate with 1 L clean filtered seawater. The *Meretrix* sp. extract was then poured into 50 mL tubes with a screw cap and kept at - 80 °C (Thermo Scientific Upright Freezer) to maintain its freshness until further use. On the day of lobster conditioning and assay, the frozen *Meretrix* sp. extract was thawed to a temperature similar to seawater in the rearing tank.

A total of 15 AA (L-alanine, L-arginine, L-serine, L-lysine, L-proline, glycine, L-asparagine, L-histidine, L-glutamine, L-threonine, L-phenylalanine, L-methionine, L-isoleucine, L-valine, leucine) and three DAA (L-glutamic acid monosodium salt monohydrate, Taurine and Betaine; all AA and DAA purchased from Sigma, St Louis, MO, USA) at three concentrations were prepared as the TS and tested individually in the present assay. Each TS was dissolved to the desired concentration with clean filtered seawater by weighing the required amount of each AA with a digital weighing scale at 0.0001 g accuracy. The TS mixture was poured into tubes with a screw cap and shaken occasionally for approximately three minutes. Each TS solution at all three concentrations was prepared prior to the behavioral assay.

### Lobster test assay conditioning

Each *T. orientalis* was conditioned to respond positively toward 10% w/v aqueous extract of the *Meretrix* sp. extract before the behavioral assay. A total of 14 *T. orientalis* (each represented a replicate) were placed individually in a 150-L white fiberglass tank (60 cm W × 60 cm L × 38 cm H) filled with 54-L (15 cm depth, surface area- 1.08 m^2^) filtered seawater. A black sunshade garden mesh net and a tray of four cm depth sand (29.5 cm W × 19.0 cm L × 4.0 cm H, surface area- 0.15 m^2^) were provided in each conditioning tank to reduce the aggression and stress in *T. orientalis*. During the lobster conditioning, the flow-through system was utilized until the daily assay was completed. The water tank system was changed back to RAS after completing the daily conditioning and behavioral assay. Each lobster was conditioned with one mL *Meretrix* sp. extract once daily at 0800 and presented with a three mL hand-held plastic pipette. The extract was injected approximately 2.0 cm in front of the antennules to reduce the dilution of the solution. All trials in conditioning and assays were video recorded with GoPro^®^ Hero9 placed approximately 15.0 cm in front of the *T. orientalis*. The recording started at least 10 s before introducing each stimulus and lasted for two minutes (Wroblewska et al., 2002). Before the conditioning and behavioral assay, the black cover mesh net of each tank was removed to ensure better video quality and presentation of each TS.

The feeding response of each *T. orientalis* towards *Meretrix* sp. extract was analyzed by replaying the recorded videotapes. Each lobster was hand-fed with fresh *Loligo* sp. at 30% of its body weight at the end of the daily conditioning and assay to compensate for its nutritional requirements. To prevent stressing the *T. orientalis*, the syphonation of unwanted particles and 30% water exchange were completed an hour before the assay every morning. The procedures of lobster conditioning with *Meretrix* sp. extract were repeated the following day until all experimental animals were well-conditioned to the positive control. When introduced with a chemoattractant, most of the crustacean species exhibited three basic chemoreceptive behaviors in the following sequence: (a) antennules flicking was initiated to identify or interpret when a chemical stimulus was introduced, (b) the feeding stimulation represented by the movement of third maxillipeds without the presence of food, and (c) the probing movements by the pereiopods to search for food that occurs if the chemical continues to exist (Liew et al., 2022; Wroblewska et al., 2002). In the present lobster conditioning, *T. orientalis* exhibited these chemoreceptive behaviors after being introduced to *Meretrix* sp. extract and consuming the administered fresh *Loligo* sp. provided later. The *T. orientalis* that demonstrated chemoreceptive behaviors to the *Meretrix* sp. extract continuously for seven days were considered to have responded positively and well-conditioned. Upon completing the conditioning, 14 *T. orientalis* were well-conditioned to *Meretrix* sp. extract with no mortality and ready for the behavioral assay. The carapace length (CL) of *T. orientalis* ranged from 50.0 to 55.0 mm. The procedures of the conditioning and behavioral assay were performed in a covered outdoor environment. Water parameters were collected and recorded once daily at 0730. During conditioning and assay, water qualities in the acclimatized tank remained at a temperature of approximately 26.27 ± 0.78 °C, 5.99 ± 0.53 mg/l dissolved oxygen (DO) levels, 7.78 ± 0.06 water pH, and 27.82 ± 0.79 ppt salinity (pH/ORD/EC/DO tester, Hanna Instruments, HI 9828).

### Behavioral assay

The procedures described in the present assay were adopted from a previous study with modifications (Zimmer-Faust, 1987). Each *T. orientalis* was introduced with only one TS at three different concentrations daily and only presented in ascending order from 10^−3^ M, 10^−2^ M to 10^−1^ M at 0800 daily. The effective concentration of the chemoattractant was estimated experimentally by adding increasing concentrations of the chemical (Lee & Meyers, 1996). TS was chosen randomly and administered in a random sequence to 14 *T. orientalis* (each represented a replicate; *n* = 14) using a hand-held pipette. The 1 ml TS was introduced to each lobster with the same description as mentioned in the previous section. While testing the different concentrations, approximately 200% of the seawater in the experimental tank was exchanged with a flow-through system to minimize the residual response and prevent desensitization of the previous TS concentration. At the end of the daily assay, each *T. orientalis* was introduced with one mL of *Meretrix* sp. extract and *Loligo* sp. to ensure the validity of tests conducted on the tested lobsters with TS. All *T. orientalis* were presented with a negative control upon completing the behavioral assay to confirm that no behavioral response towards TS was elicited from the seawater.

### Data collection and analysis

The behavioral responses of lobsters towards each chemical stimulus were analyzed, with the classification of behavioral scores designed based on the chemoreceptive behaviors towards the *Meretrix* sp. extract (see Table 1). Behavioral responses of *T. orientalis* to each stimulus concentration within two minutes were scored based on the four possible outcomes: (i) score 0 = unresponsive *T. orientalis*, (ii) score 1 = *T. orientalis* with an increment of antennular flicking, and (iii) score 3 = the frequent movement of third maxillipeds rubbed each other and accompanied with movement antennular flicking (score 1), and lastly, (iv) score 3 was allocated when the lobster demonstrated ground probing with first and second pereiopods with antennular flicking (score 1) and movement of maxillipeds (score 2). Subsequently, the mean of the response score of each chemical substance was calculated and displayed as the representative outcome. All statistical tests were performed using the R studio (PMCMR package). Two statistical analyses were conducted based on the mean scores of the repeated samples: (i) different TS of each concentration, and (ii) different concentrations of each TS. The mean of both statistical analyses was analyzed with the non-parametric repeated measures Friedman Test. The Conover post hoc test was performed when a significant difference (*p* < 0.05) was found among the treatments, and the Bonferroni correction was applied to minimize Type I error.

**Table 1:** Classification of the behavioral responses of *T. orientalis* towards TS.

**Phase**	**Behavioral responses**	**Scores**
I	No response	0
II	Increment in antennular flicking rate	1
III	Frequent movement of third maxillipeds rubbed each other accompanied by movement (II)	2
IV	Probing the ground with first and second pereiopods together with behaviors (II) and (III)	3

## Results

### Behavioral response score

#### Different TS at each concentration

**Figure 1:**
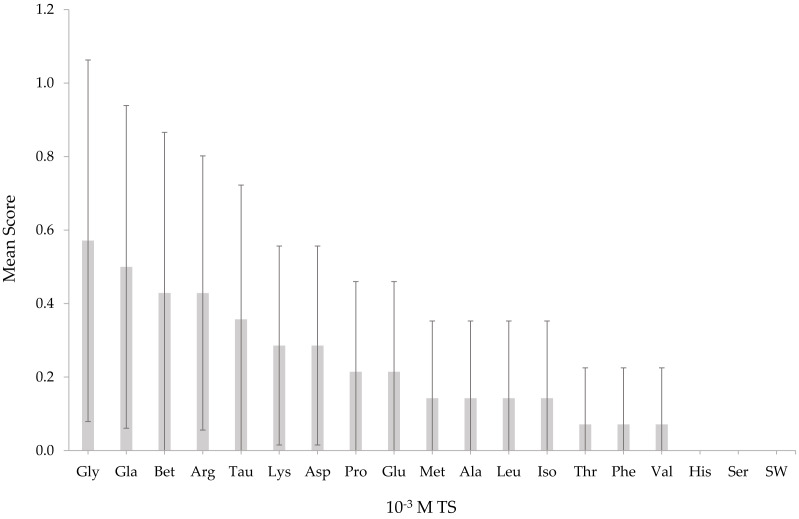
Mean response score of wild-caught *T. orientalis* towards 10^−3^ M test stimuli (TS) and SW. Different superscript alphabets indicate significant differences at *P* < 0.05 (number of replicate *n* = 14); vice versa. Vertical bars indicate standard error. Gly, Glycine; Gla, L-glutamic acid monosodium salt monohydrate; Bet, Betainel Arg, L-arginine; Lys, L-lysine; Asp, L-asparagine; Tau, Taurine; Pro, L-proline; Glu, L-glutamine; Met, L-methionine; Ala, L-alanine; Leu, Leucine; Iso, L-isoleucine; Thr, L-threonine; Phe, L-phenylalanine; Val, L-valine; His, L-histidine; Ser, L-serine. All chemicals were purchased from Sigma (St Louis, MO, USA).

**Figure 2:**
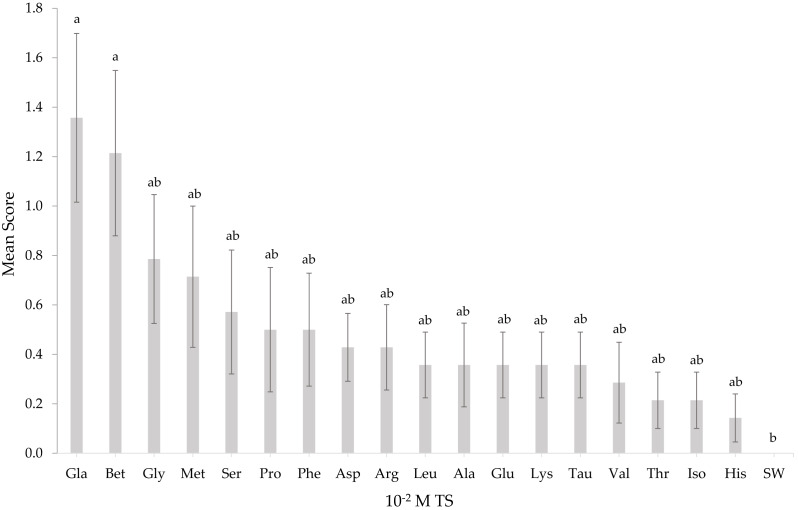
Mean response score of wild-caught *T. orientalis* towards 10^−2^ M test stimuli (TS) and SW. Different superscript alphabets indicate significant differences at *P* < 0.05 (number of replicate *n* = 14). Vertical bars indicate standard error.

**Figure 3:**
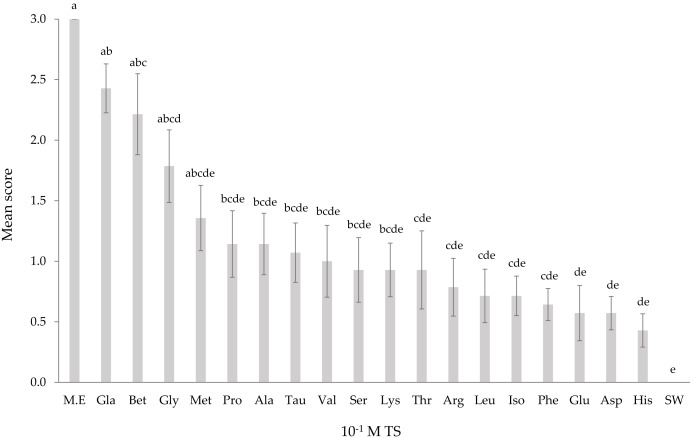
Mean response score of wild-caught *T. orientalis* towards 10^−1^ M test substances (TS) and SW. Different superscript alphabets indicate significant differences at *P* < 0.05 (number of replicate *n* = 14). Vertical bars indicate standard error. M.E = *Meretrix* sp. extract.

*T. orientalis* responded to TS solutions at a concentration of 10^−3^ M (Fig. 1) except for histidine and serine. At the lowest concentration (10^−3^ M), none of the mean response scores from all TS solutions elicited any significant differences (*p* > 0.05). All TS tested on *T. orientalis* did not significantly differ (*p* > 0.05) from SW as the concentration elevated to 10^−2^ M (Fig. 2), except for L-glutamic acid monosodium salt monohydrate and betaine. Upon increasing the TS concentration to 10^−1^ M (Fig. 3), the L-glutamic acid monosodium salt monohydrate reflected the strongest responses in comparison to other tested stimuli. *T. orientalis* demonstrated a perfected mean behavioral response (100%) when exposed to the *Meretrix* sp. extract (positive control) than all TS tested at 10^−1^ M concentration, which indicates that this extract was the most attractive chemical solution. However, no significant differences (*p* > 0.05) were observed among the mean response scores of positive control, L-glutamic acid monosodium salt monohydrate, betaine, glycine, and L-methionine. At 10^−1^ M concentration, the lobsters disclosed a significantly higher (*p* < 0.05) mean behavioral response score to L-glutamic acid monosodium salt monohydrate, betaine, glycine, and L-methionine than the other 14 TS. In contrast, the lobsters exhibited the lowest mean behavioral response score to L-histidine among all TS, but only significantly lower (*p* < 0.05) than the positive control, L-glutamic acid monosodium salt monohydrate, and betaine. The confirmation that a higher mean response score of *T. orientalis* was contributed by the TS and positive control is evident in the significant differences (*p* < 0.05) observed between the negative control (SW), positive control, L-glutamic acid monosodium salt monohydrate, betaine and glycine at 10^−1^ M concentration.

#### Different concentrations at each TS

**Figure 4:**
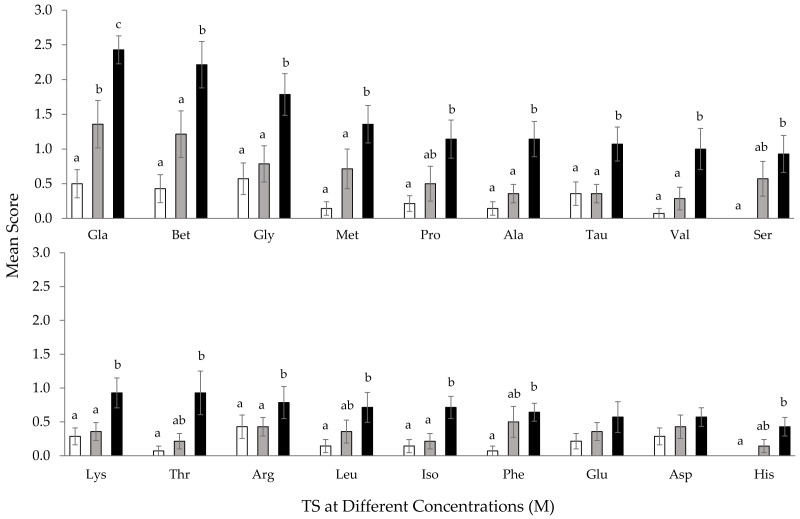
Comparison of mean response score of wild-caught *T. orientalis* towards 10^−3^ M (empty bar), 10^−2^ M (grey bar) and 10^−1^ M (black bar) concentrations of each test stimuli (TS). Different superscript alphabets indicate a significant difference between concentrations of each TS at *P* < 0.05. Vertical bars indicate standard error.

The behavioral activity of *T. orientalis* increased in line with elevated TS concentrations (Fig. 4). Increased response scores were observed when *T. orientalis* was exposed to most of the 10^−2^ M TS. A significant increase (*p* < 0.05) was only observed in L-glutamic acid monosodium salt monohydrate at 10^−2^ M concentration. The mean response scores to 10^−2^ M L-glutamic acid monosodium salt monohydrate and betaine increased further by 171% and 143% compared to 10^−3^ M concentration, respectively. A further increase in concentration to 10^−1^ M AA elicited a significantly higher (*p* < 0.05) mean response score for all TS, excluding L-proline, L-serine, L-threonine, leucine, L-phenylalanine, L-asparagine, L-glutamine, and L-histidine. The highest significant response was also observed in L-glutamic acid monosodium salt monohydrate (*p* < 0.05). Notably, no significant effect was recorded for the mean response score of *T. orientalis* towards L-glutamine and L-asparagine (*p* > 0.05) to various concentrations. The rank order of the mean score was affected by the concentration of test stimulus but the most potent stimulatory AA at 10^−1^ M concentration was similar to 10^−2^ M TS. At the highest concentration, the behavior response scores of L-glutamic acid monosodium salt monohydrate and betaine reflected an increment of 79% and 82% than the 10^−2^ M concentration.

### Behavioral activities

**Figure 5:**
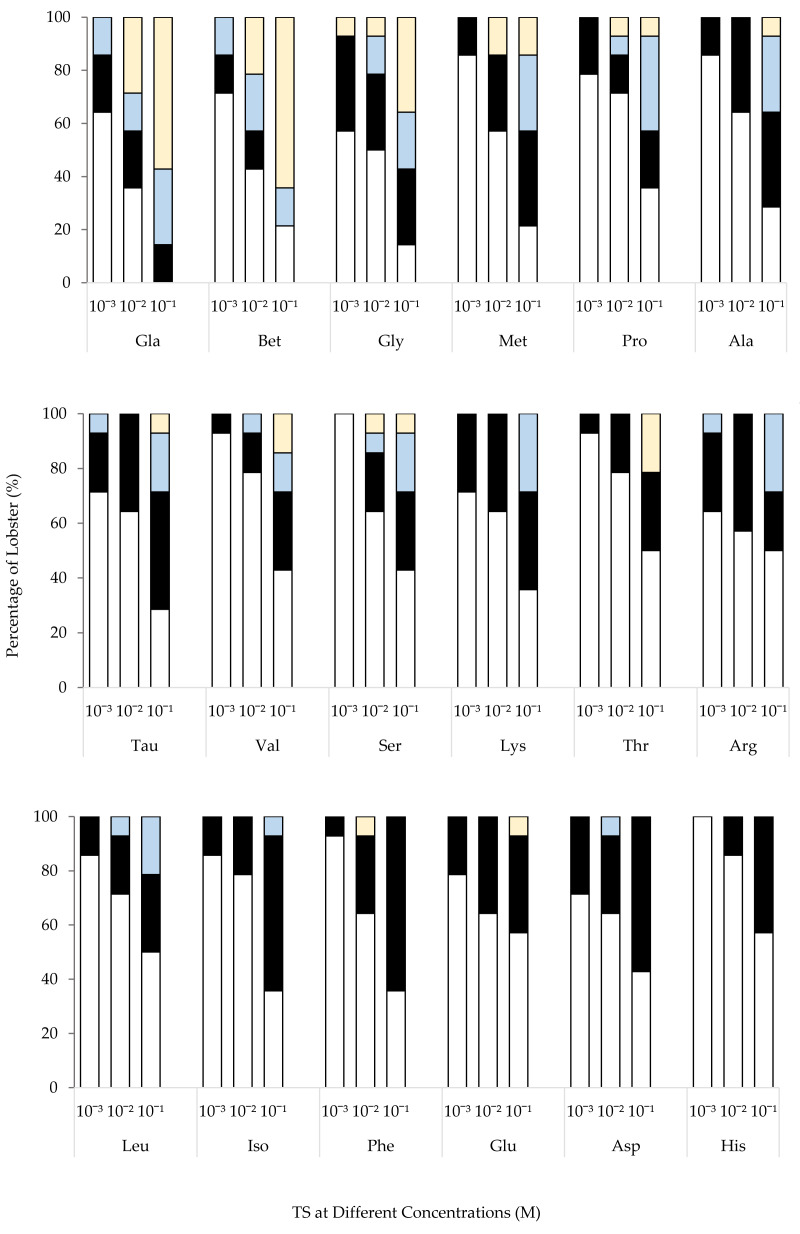
Percentage of *T. orientalis* with different behavioral response categories (0, 1, 2, and 3) at different concentrations (10^−3^, 10^−2^ and 10^−1^ M) of each test substance (TS).

Despite using a low concentration (10^−3^ M), glycine was the only TS that successfully elicited a probing response in a lobster (see Fig. 5). Frequent movement of third maxillipeds was observed in lobsters introduced with L-glutamic acid monosodium salt monohydrate, betaine, taurine, and L-arginine. Meanwhile, 11 TS (L-proline, L-methionine, L-alanine, L-leucine, L-glutamine, L-lysine, L-threonine, L-phenylalanine, L-isoleucine, L-asparagine, and L-valine) tested were only categorized by antennular flicking. Similar to SW (negative control), the lobsters failed to elicit any behavioral response to histidine and serine at 10^−3^ M concentration. This result suggests that the detection of histidine and serine is restricted at low concentrations. As the concentration increased to 10^−2^ M, a higher probing response was promoted by TS, which includes L-glutamic acid monosodium salt monohydrate, betaine, glycine, L-methionine, L-proline, L-serine, and L-phenylalanine. While L-valine, L-leucine, and L-asparagine induced at least a second phase response on a *T. orientalis*. The remaining TS only elicited antennular flicking at best. *T. orientalis* strongly responded to 10^−2^ M L-glutamic acid monosodium salt monohydrate compared to other TS.

At 10^−1^ M concentration, probing behavioral response observed from *T. orientalis* was elicited by 61% of TS tested (L-glutamic acid monosodium salt monohydrate, betaine, glycine, L-methionine, L-proline, L-alanine, taurine, L-valine, L-serine, L-threonine, and L-glutamine). Although betaine reflected the highest probing response, 21% of *T. orientalis* did not elicit any responses at this relatively high concentration of 10^−1^ M. In contrast, 10^−1^ M L-glutamic acid monosodium salt monohydrate was the only TS that successfully elicited behavioral responses in all *T. orientalis*. Among these test substances, L-lysine, L-arginine, L-leucine, and L-isoleucine triggered at least two response phases. The remaining TS (L-phenylalanine, L-asparagine, and L-histidine) only induced antennules flicking at most. Furthermore, the antennular grooming behavior of *T. orientalis* was almost exclusively activated by L-glutamic acid monosodium salt monohydrate, particularly after an increased concentration.

## Discussion

The present study is the first report on the potent stimulatory chemoattractant of *T. orientalis* for different concentrations of AA. Overall, the behavioral assay depicted that L-glutamic acid monosodium salt monohydrate, betaine, and glycine were the most potential stimulatory substances for *T. orientalis*. These substances are commonly present in natural food and abundant in marine invertebrate flesh (Ache, Gleeson & Thompson, 1988; Tolomei, Crear & Johnston, 2003; Zimmer-Faust, 1991) and are promising chemoattractants for *Jasus edwardsii* (Tolomei, Crear & Johnston, 2003), *P. interruptus* (Zimmer-Faust, 1991), *Penaeus monodon* (Coman et al., 1996) and crab *Erimacrus senbeckii* (Takei, 1977). Unique differences were also detected by Fuzessery & Childress (1975) when spiny lobster *P. interruptus* was tested with either monocarboxylic or dicarboxylic amino acids. Nonetheless, contradicting results were reported in spiny lobsters. *Homarus americanus* (Carter & Steele, 1982) exhibited positive responses towards L-proline, L-arginine, L-lysine, L-alanine, glycine, and L-valine in descending trend, whereas *P. argus* (Carter & Steele, 1982) preferred taurine and glycine, and *P. interruptus* (Zimmer-Faust, 1987; Zimmer-Faust et al., 1984) was highly elicited by glycine, L-alanine, and L-serine. Thus, the present results support the species-specific characteristic of crustaceans in response to AA.

L-glutamic acid monosodium salt monohydrate achieved the highest mean score in *T. orientalis* among the potent stimulatory chemoattractants explored in this study. L-glutamic acid monosodium salt monohydrate exclusively activated the antennular grooming behavior (AGB) responses of *T. orientalis*, thus suggesting L-glutamate and the L-glutamic acid monosodium salt monohydrate may have comparable chemosensory properties. L-glutamate is the only chemical that effectively elicited AGB in Panulirus (Barbota & Daniel, 1997; Cericola & Daniel, 2010; Wroblewska et al., 2002). According to Schmidt & Derby (2005), AGB in lobster consists of a stereotyped movement sequence and is distinguishable from all other behaviors. AGB stems from the sticky nature of glutamate and is easily detected with spiny lobsters’ chemoreceptors (Barbota & Daniel, 1997). Apart from cleaning and resetting chemoreceptors in antennules (Snow, 1973), Fuzessery & Childress (1975) highlighted that antennular grooming played a role in transferring stimulus molecules to mouthparts. Since AGB was used as an indicator of appetitive feeding in *P. interruptus* (Zimmer-Faust et al., 1984), supplementing L-glutamic acid monosodium salt monohydrate in the practical diet for *T. orientalis* might resolve the constraints faced in this preliminary experiment.

As an ionic form of glutamate, glutamic acid had been reported as the predominant taste-active constituent of typical food extract (Barbota & Daniel, 1997). It is one of the major taste-active-free AA found in *Meretrix meretrix* (Chen et al., 2012) and *M. lusoria* (Karnjanapratum et al., 2013). *Thenus* spp. exhibited remarkable affinity towards mollusks, and *T. orientalis* appears to consume cuttlefish and squid in the wild (Lavalli, Spanier & Grasso, 2007). Our preliminary study on the daily feeding activities of *T. orientalis* revealed that the lobster expressed a noticeable preference for *Meretrix* sp. over *Loligo* spp. and *Polymesoda expansa*. However, no feeding was observed when introduced with *Decapterus* sp. and *Litopenaeus vannamei*. L-glutamic acid monosodium salt monohydrate can be suggested as an essential feeding signal for this species considering its high concentration in the preferable food for *T. orientalis*.

Betaine and glycine are chemicals commonly found in high concentrations in prey tissue (Fuhrman & Bell, 1985). Both substances were widely used in previous stimulation experiments of spiny lobsters (Tolomei, Crear & Johnston, 2003; Zimmer-Faust, 1991; Zimmer-Faust, 1987; Zimmer-Faust et al., 1984). Additionally, the popularity of glycine as a test substance was due to its abundance in coastal seawater (Fuhrman & Bell, 1985; Tada, Tada & Maita, 1998; Yusenko et al., 2008). Studies reported that betaine and glycine successfully stimulated specific chemoreceptors neurons in *P. argus* (Ache, Gleeson & Thompson, 1988), *P. interruptus* (Zimmer-Faust, 1991), and *P. monodon* (Coman et al., 1996). Moreover, glycine was reported as one of the most stimulatory substances in *H. americanus* (Derby & Atema, 1982). Sheppard (Sheppard, 2001) found that glycine significantly improved the feed intake of *J. edwardsii* compared to taurine and betaine when incorporated into a formulated diet.

Concentration is one of the chemical cue properties that contribute to stimulating behavioral responses in crustaceans (Zimmer-Faust, 1991). In the present study, a higher concentration of L-glutamic acid monosodium salt monohydrate, betaine, and glycine effectively and significantly stimulated and increased behavioral responses ( *p* < 0.05) in *T. orientalis*. Likewise, Barbota & Daniel (1997) and Wroblewska et al. (2002) also reported that the behavioral responses of *P. argus* exposed to a lower concentration of betaine and glycine were ineffective compared to higher concentrations. The antennular flicking rate in *P. interruptus* was catalyzed with increasing glycine concentrations (Zimmer-Faust, 1991). Similarly, lower behavioral activities were observed in *P. monodon* presented with lower concentrations of glutamine, glycine, and betaine below 10^−6^ M compared to higher concentrations (Coman et al., 1996). A significant standard error in the present results might be linked to the heterogeneous population of chemoreceptors in *T. orientalis* (Johnson & Ache, 1978; Lavalli, Spanier & Grasso, 2007).

None of the test substances, including L-glutamic acid monosodium salt monohydrate attained a response score as observed in clam extract. All *T. orientalis* introduced with *Meretrix* sp. extract recorded maximum scores, which probed the ground with pereiopods together with frequent movement of antennules and third maxillipeds. This finding parallels other studies on spiny lobsters (Coman et al., 1996; Fuzessery & Childress, 1975; Heinen, 1980) and crab *Carcinus maenas* (Shelton & Mackie, 1971). Reportedly, the behavioral activity of a mixture was higher than single AA in crustaceans (Fuzessery & Childress, 1975; Heinen, 1980). The mixture of AA activates several receptor sites with a different compound in the mixture itself (Coman et al., 1996), whereas single AA single mostly competes for receptor sites (Fuzessery & Childress, 1975). Zimmer-Faust et al. (1984) found that the feeding and locomotor behavior of *P. interruptus* to chemical extracts was almost identical to the whole food. The presence or absence of this AA in the natural diet depicted apparent differences in excitatory capability in each chemical (Tolomei, Crear & Johnston, 2003). Glutamate and glycine appeared to be stimulatory single substances, whereas the mixture of betaine with other AA was more stimulated than being tested individually (Heinen, 1980). Betaine may be synergetic with dietary AA, thus mixing betaine alone with dietary AA might yield promising results (Heinen, 1980). Further studies are necessary to elucidate this hypothesis.

## Conclusions

Summarily, L-glutamic acid monosodium salt monohydrate, betaine, and glycine were the most potent stimulatory chemoattractant AA investigated in this study. L-glutamic acid monosodium salt monohydrate stimulated the highest responses significantly (*p* < 0.05) in *T. orientalis* at 10^−1^ M concentration than 15 other test substances and negative control. Further studies are required to explore the AA mixture as a feeding stimulant rather than a single AA.

## Additional Information and Declarations

### Field Study Permissions

The following information was supplied relating to field study approvals (i.e., approving body and any reference numbers): The Sabah Biodiversity Council approved the experimental animals used in this study (Licence Ref.No. JKM/MBS.1000-2/2 JLD.13 (96)). The animal study protocol was approved by the JKEHUMS Universiti Malaysia Sabah Animal Ethics Committee (Approval number: AEC008/2022).

### Data Availability

The following information was supplied regarding data availability: The data is available at figshare: Teoh, Chui Fen; Tuzan, Audrey Daning (2022). Behavioural Responses of the Slipper Lobster Thenus orientalis towards Crystalline Amino Acids. figshare. Media. https://doi.org/10.6084/m9.figshare.21009184.v1.
